# Supplementary material for: Labeled TEMPO-Oxidized Mannan Differentiates Binding Profiles within the Collectin Families
Source: Int J Mol Sci. 2022 Dec 16;23(24):16067. doi: 10.3390/ijms232416067 (PMC9786299; doi:10.3390/ijms232416067)
Supplement: Supplementary file 1 [file ijms-23-16067-s001.zip › ijms-2042914-supplementary.pdf]

Labeled TEMPO-oxidized mannan differentiates binding profiles within the collectin families.

Florent Le Guern [1,2], Anne Gaucher [1], Gina Cosentino [2], Marion Lagune [2], Henk P. Haagsman [3], Anne-Laure Roux [4,5], Damien Prim [1] and Martin Rottman [2,4,5,*]

1 Institut Lavoisier de Versailles, CNRS, UVSQ, Université Paris-Saclay, 78035 Versailles, France ; anne.gaucher@uvsq.fr (A.G.) ; damien.prim@uvsq.fr (D.P.)
2 Faculté de Médecine Simone Veil, Université de Versailles St Quentin, INSERM UMR U1173, 2 Avenue de la source de la Bièvre, 78180 Montigny le Bretonneux, France ; g.cosentino@hotmail.fr (G.C.) ; marion.lagune@uvsq.fr (M.L.) ; martin.rottman@aphp.fr (M.R.)
3 Section Molecular Host Defence, Division Infectious Diseases & Immunology, Department of Biomolecular Health Sciences, Faculty of Veterinary Medicine, Utrecht University, Utrecht, the Netherlands; H.P.Haagsman@uu.nl (H.P.H.)
4 Hôpital Raymond Poincaré, AP-HP, GHU Paris Saclay, 104 Bd Poincaré, 92380 Garches, France ; anne-laure.roux@aphp.fr (A-L.R)
5 Plateforme des biomarqueurs innovants, 104 Bd Poincaré, 92380 Garches, France

* Correspondence: martin.rottman@aphp.fr

Figure S1. IR spectra of **Man, Mox, Mf** 2
Figure S2. NMR ^{13}C of **Mox 24%** 3
Figure S3. SEC-MALS analysis of **Man** and **Mox 24%** 4
Figure S4. NMR ^{1}H analysis of **2** 4
Figure S5. NMR ^{1}H analysis of **3** 5
Figure S6. NMR ^{1}H analysis of **4** 5
Figure S7. Absorbance, fluorescence spectra and quantum yields of **4** and fluorescein in NaOH 0.1M. Fluorescein was chosen as the reference for quantum yields determination, since its yield is already described (J.R. Lakowicz, Principles of Fluorescence Spectroscopy, 2nd Ed., Kluwer Academic/Plenum Publishers, New York, London, Moscow, Dordrecht, 1999.) 6

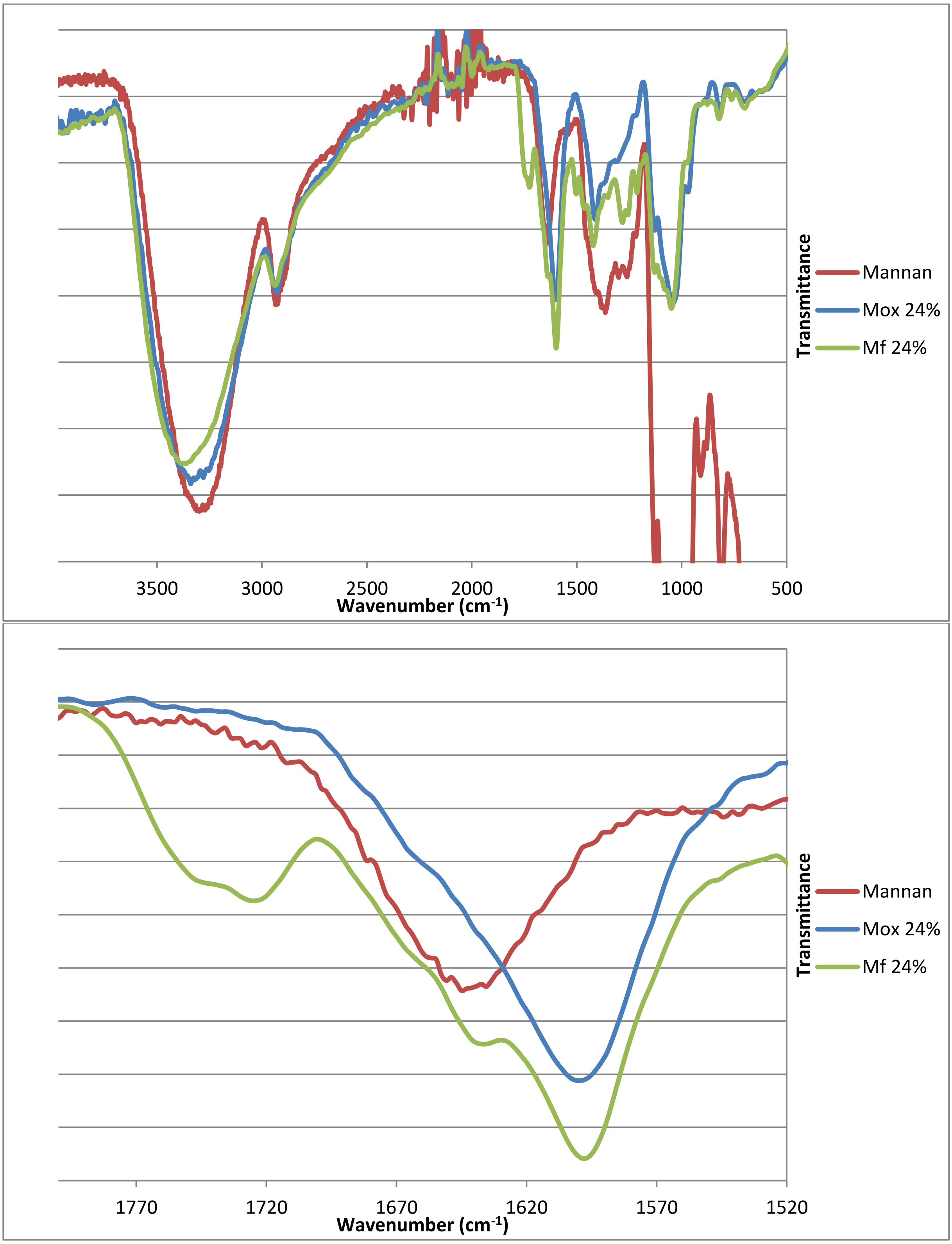

Figure S1. IR spectra of **Mannan, Mox, Mf**

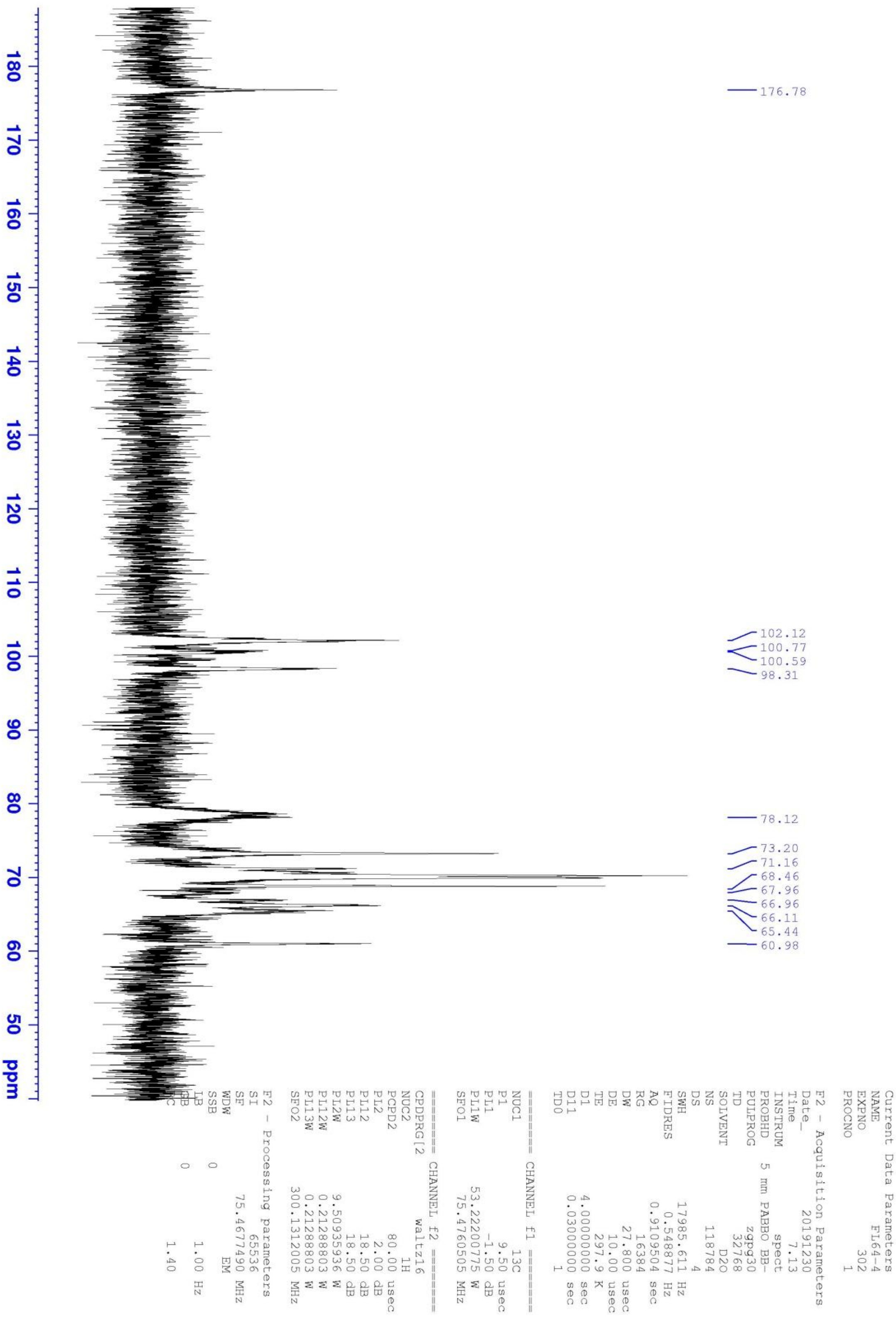

Figure S2. NMR ^{13}C of **Mox 24%**

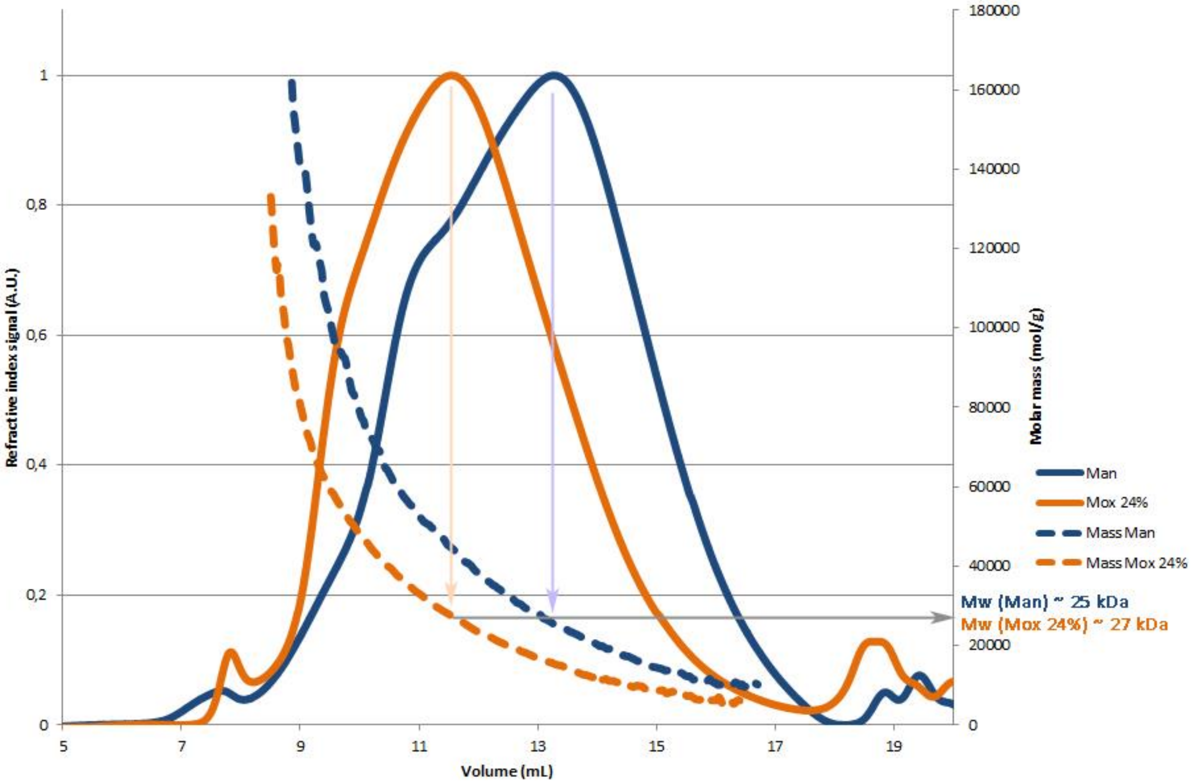

Figure S3. SEC-MALS analysis of **Mannan (Man)** and **Mox 24%**

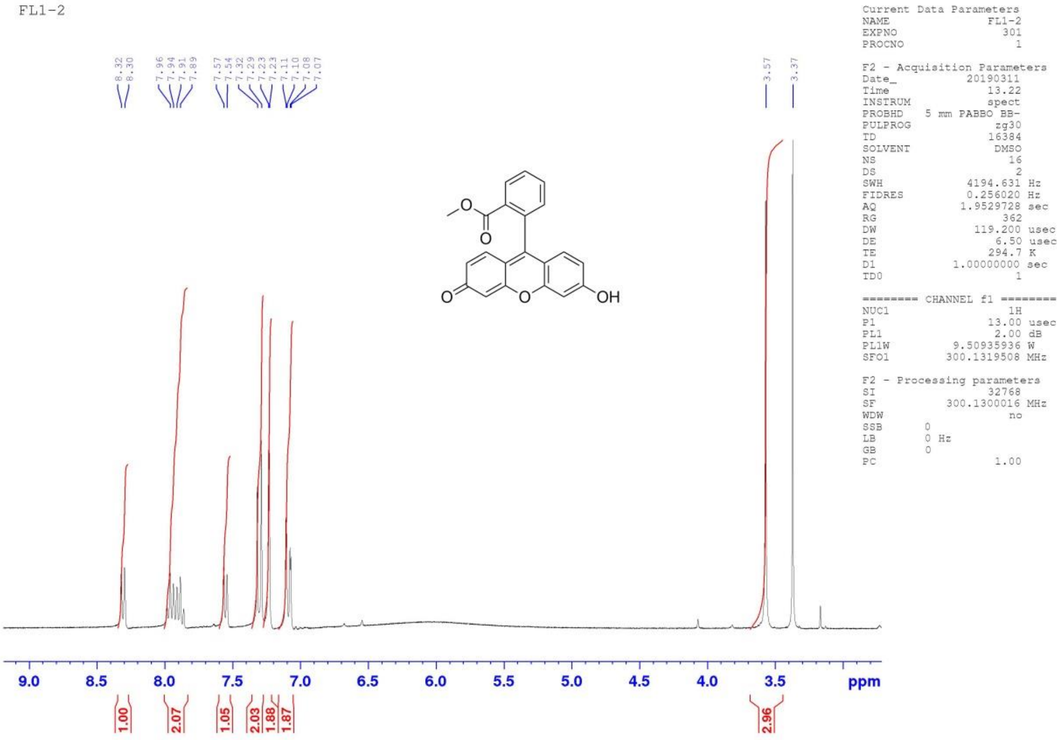

Figure S4. NMR ^{1}H analysis of **2**

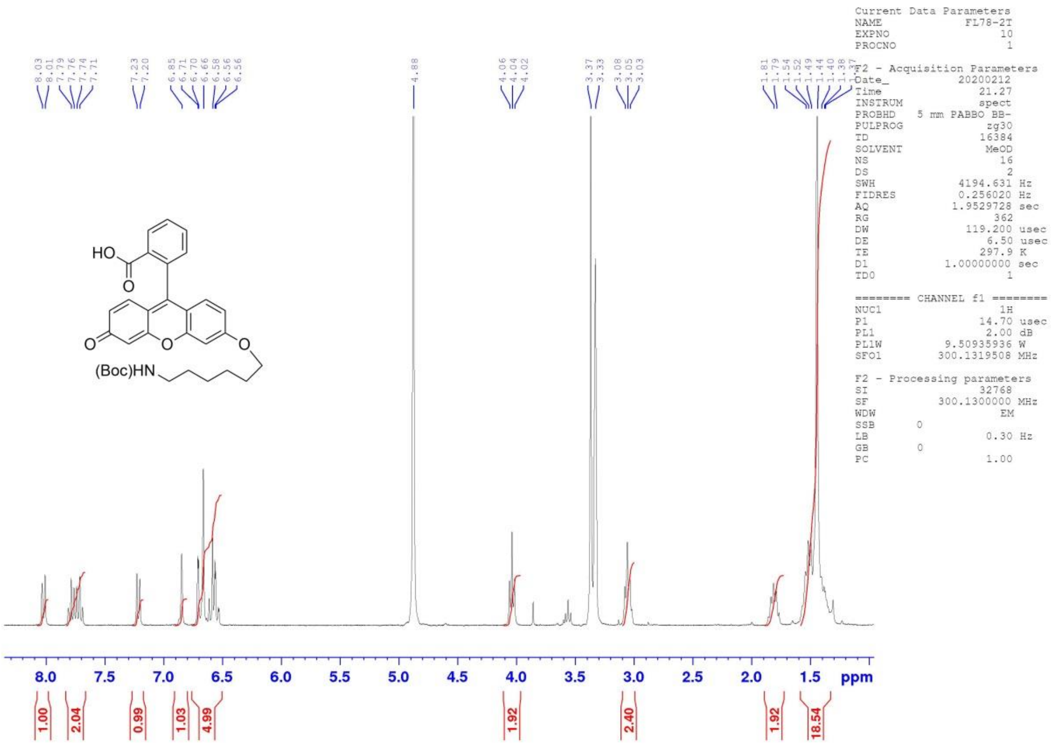

Figure S5. NMR ^{1}H analysis of **3**

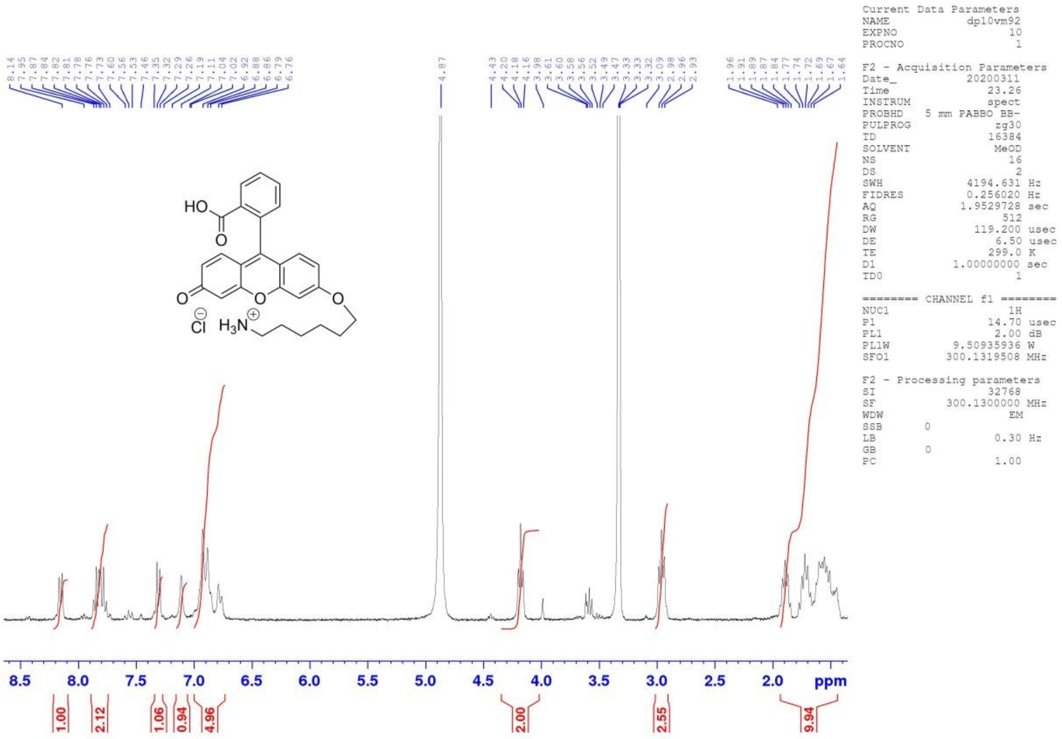

Figure S6. NMR ^{1}H analysis of **4**

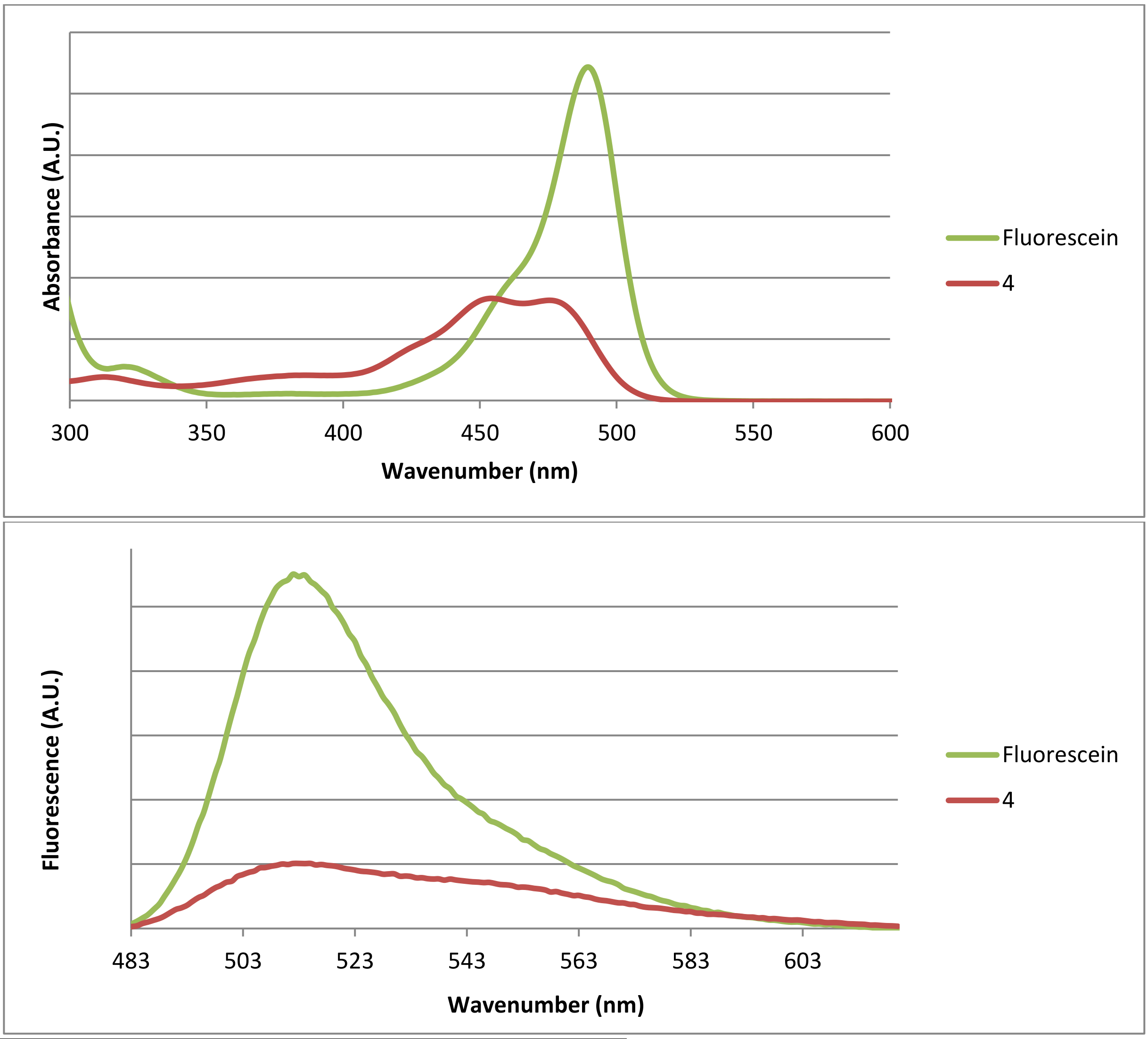

	λ (nm)	Φ_{f*}
Fluorescein	490	0,95
**4**	475	0,32

Figure S7. Absorbance, fluorescence spectra and quantum yields of **4** and fluorescein in NaOH 0.1M. Fluorescein was chosen as the reference for quantum yields determination, since its yield is already described (J.R. Lakowicz, Principles of Fluorescence Spectroscopy, 2nd Ed., Kluwer Academic/Plenum Publishers, New York, London, Moscow, Dordrecht, 1999.)
